# An engineered hydrogel with low-dose antitumor drugs enhances tumor immunotherapy through tumor interstitial wrap

**DOI:** 10.3389/fbioe.2022.1072393

**Published:** 2022-11-14

**Authors:** Zhongxian Li, Jiawei Xiang, Qiang Zhang, Mingyuan Zhao, Yuan Meng, Jie Zhong, Tingting Li, Lanxin Jia, Kai Li, Xi Lu, Zhuo Ao, Dong Han

**Affiliations:** ^1^ CAS Center for Excellence in Nanoscience, National Center for Nanoscience and Technology, Beijing, China; ^2^ University of Chinese Academy of Sciences, Beijing, China; ^3^ Hebei Key Lab of Nano-Biotechnology, Hebei Key Lab of Applied Chemistry, Yanshan University, Qinhuangdao, China; ^4^ College of Life Sciences, Bejing University of Chinese Medicine, Beijing, China; ^5^ CAS Key Laboratory of Bio-Inspired Materials and Interfacial Science, Technical Institute of Physics and Chemistry, Chinese Academy of Sciences, Beijing, China

**Keywords:** immunogenic cell death, interstitial administration, hydrogel, tumor immunotherapy, tumor microenvironment

## Abstract

Stimulating immunogenic cell death (ICD) is the key to tumor immunotherapy. However, traditional chemoradiotherapy has limited effect on stimulating immunity and often requires repeated administration, which greatly reduces the tumor-killing effect. In this article, we created a sodium alginate hydrogel sustained-release system containing low-dose doxorubicin (Dox) and immune adjuvant R837, which were injected into the interstitial space to wrap around the tumor *in situ*, achieving a sustained release and long-lasting immune response. Cooperating with immune checkpoint blockade, Dox induced ICD, activated dendritic cells (DCs) and converted immunosuppressive M2-type tumor-associated macrophages (TAM) to tumor-killing M1-type TAMs. Simultaneously, it greatly promoted T cell proliferation and infiltration, and reduced tumor immunosuppressive factors, triggering a robust immune response to suppress tumors *in vivo*. In conclusion, this anti-tumor strategy based on interstitial injection can achieve continuous local immune stimulation by low-dose chemotherapy drugs, providing a potential approach for tumor immunotherapy.

## Introduction

As a process of activating the immune system and triggering a robust immune response to promote the tumor necrosis, tumor immunotherapy includes immune checkpoint inhibitors, adoptive cellular therapy, and tumor vaccines (Zhang et al., 2019; Zhang and Zhang, 2020). It is a beneficial supplement to traditional surgery, and chemoradiotherapy, significantly improving anti-tumor efficiency and prolonging patient survival (Waldman et al., 2020). Dying tumor cells can produce damage-associated molecular patterns (DAMPs) that are exposed on the cell surface, promote DC maturation, then T cells and memory T cells are recruited, which infiltrate into tumors and trigger tumor immunogenic cell death (ICD) (Kroemer et al., 2013; Galluzzi et al., 2020; Jin and Wang, 2021). Initiation of ICD can convert tumor environment from a “cold” (immunosuppressive) state into a “hot” (immune-responsive) state, which causes tumor regression of both *in situ* and distant metastases (Dewan et al., 2009; Zhao et al., 2022).

Although chemotherapy or radiotherapy can induce the ICD process (Krysko et al., 2012; Rios-Doria et al., 2015), their immune responses are not strong enough. Thus, additional auxiliary stimulation is often needed to amplify the immune response, such as breaking the reactive oxygen species balance in tumor tissues (Aboelella et al., 2021), cold tumor heating (Mansurov et al., 2020), scaled delivery of chemotherapy drugs (Wang et al., 2021; Zhang et al., 2021), photothermal drug combined therapy (Liu et al., 2022a) so as to enhance the anti-tumor effect and shorten the cycle of chemotherapy. However, these strong stimuli inevitably trigger side effects of cancer immune responses, such as myelosuppression, lymphopenia, and other inflammatory responses (Kepp et al., 2020; Banstola et al., 2021; Kroemer et al., 2022). Due to the highly immunosuppressive and heterogeneous microenvironment of tumors, the ability to release tumor-associated antigens is weakened, and a timely and effective immune response cannot be generated, resulting in continuous tumor growth (Ngambenjawong et al., 2017; Duan et al., 2018; Musetti and Huang, 2018). Therefore, ICD with lower stimulation to enhance the immune response and modulate the immune system to improve the tumor microenvironment is crucial for more efficient tumor immunotherapy.

In previous studies, it has been found that interstitial fluid filled space enables flattened connections between functional structures in organisms, which show promise for improving disease pathophysiology (Foster and McVey Neufeld, 2013; Budden et al., 2017; Liu et al., 2022b). Interstitial drug delivery showed the effects of activating and recruiting immune cells for better clearance of parasites resident in organs, and accelerated pathological recovery (Zhang et al., 2020a). From the damaged area to the tumor area, the liquid metal moved through and wrapped around the tumor, separating the tumor from the surrounding normal vessel supply (Hu et al., 2018; Cao et al., 2020). With the help of interstitial drug delivery, the toxicity caused by intravenous administration can be avoided, and low-dose chemotherapy drugs can be wrapped around the tumor tissues and released slowly, providing a sustained tumor-killing effect (Xu et al., 2020). This can ensure the possibility for the efficient presentation of tumor antigens from dying tumor cells to lymph node DCs (Zhang et al., 2020b). However, in the current tumor therapy, there are few studies on using the interstitial structure to assist in enhancing the immune response to achieve anti-tumor therapy. Therefore, developing a novel drug delivery strategy *via* the interstitial route to trigger ICD will bring inspiration for tumor immunotherapy.

Here, we prepared an injectable sodium alginate hydrogel as a sustained-release system carrying the hydrophobic immune adjuvant R837 and low-dose Dox. The drug-loaded hydrogel was injected into the interstitial space around the tumor tissues, which produced an effective wrap against tumor growth, invasion and metastasis. Firstly, the interstitial blocking restricts the nutrition supply in tumor sites from normal tissues, which physically limited tumor growth. What’s more, the gel slowly released Dox to inhibit tumor growth while triggering an immune response, and low-dose Dox was enough to induce ICD with low system toxicity, while the supplement loading of R837 in the hydrogel can effectively amplify the immunogenicity. We obtained a better anti-tumor efficacy when combined with immune checkpoint inhibitor, aPD-L1. Wrapping tumor tissues by the interstitial route can inhibit tumor invasion and metastasis, Dox induced ICD can achieve rapid antigen presentation. R837 adjuvant could better stimulate tumor immunotherapy, greatly promote T cell infiltration and activation, and significantly reduce immunosuppressive factors in tumor microenvironment (Lei et al., 2022; Wang et al., 2022). Therefore, this anti-tumor strategy based on interstitial injection with low-dose chemotherapeutic drugs, enhancing immune response, provides a feasible solution for tumor immunotherapy.

## Materials and methods

### Materials, animals and antibodies

Sodium alginate was purchased from Sigma-Aldrich (United States); anhydrous calcium chloride was purchased from Xilong Chemical; doxorubicin was purchased from Rhawn Co., Ltd. (Shanghai, China); IMIQUIMOD (R837) was purchased from Macklin Biochemical Co., Ltd. (Shanghai, China); Durvalumab (anti-PD-L1) was purchased from Merck, Inc. (United States); Viability/Cytotoxicity Assay Kit was purchased from Mei5 Biotechnology Co., Ltd.; TNF-α Elisa Kit and IL-6 Elisa Kit was purchased from MEIMIAN Inc. (Jiangsu, China); Cell Counting Kit-8 was purchased from Beijing Solarbio Science & Technology Co., Ltd. (Beijing, China); Anti-mouse CD45-Alexa Fluor^®^ 700, anti-CD80-Percp, anti-CD11b-FITC, anti-CD80-FITC, anti-H-2Kd-PE, anti-CD83-FITC, anti-CD206-APC, anti-F4/80-APC, anti-CD86-PE, anti-CD3-FITC, anti-CD4-Percp, anti-CD8-PE were purchased from BioLegend, Inc. (United States).

The murine melanoma cell line B16F10 was obtained from the Cell Bank of the Chinese Academy of Sciences (Shanghai, China). B16F10 cells were cultured in RPMI 1640 medium supplemented with fetal bovine serum (FBS, 10%, v/v, Gibco, United States) and antibiotic-antifungal solution (1%, v/v, Gibco, United States), 37°C and 5% CO_2_ atmosphere. All experiments were performed using cells in logarithmic growth phase. Eight-week-old male C57BL/6 mice were obtained from Beijing Vital River Laboratory Animal Technology Co., Ltd. (Beijing, China).

All animal handling procedures followed ethics committee guidelines of National Center for Nanoscience and Technology.

### Gelling amount

At room temperature, 375 mg of sodium alginate powder was dissolved in 30 ml of 0.9% sodium chloride injection, and stirred overnight to fully dissolve the sodium alginate to prepare a 12.5 mg/ml sodium alginate solution. Take 2 ml of the prepared 12.5 mg/ml sodium alginate solution and add it to a 5 ml capacity vial. Calcium chloride solutions with concentrations of 2, 3, 4, 4.5, 5, 5.5, and 6 mg/ml was prepared with anhydrous calcium chloride, and the solvent was 0.9% sodium chloride injection. Finally, 0.5 ml of calcium chloride solution was added to sodium alginate solution while stirring. Invert each bottle and observe the minimum calcium chloride concentration required for gel formation.

### Preparation of Alg-Dox-R837 hydrogel system

We took 2 ml of the 12.5 mg/ml sodium alginate solution prepared by the above method which was accompanied by Dox and R837, and added 0.5 ml of 5 mg/ml calcium chloride solution. Finally, the mixture was stirred so that the final Dox concentration was 0.5 mg/ml and the concentration of R837 was 1 mg/ml to form a stable Alg-Dox-R837 hydrogel system. Subsequent drug-laden hydrogel systems were prepared in the same way.

### Morphological characterization

The surface morphologies of Alg, Alg-Dox and Alg-Dox-R837 xerogel samples were characterized by SEM (Hitachi S4800 + EDS, Hitachi, Japan). The xerogels were first dehydrated by a freeze-drying device, and after sample preparation, platinum was coated with ion sputter coater (Lab 18, Lesker). The image of the gel morphology was observed at an accelerating voltage of 10 kV. The composition of the elements on the surface of the gel was observed utilizing energy spectroscopy.

### Rheological testing of drug-loaded hydrogels and injectability

Alg, Alg-Dox and Alg-Dox-R837 hydrogel samples were determined using a dynamic mechanical rheological stress meter (Haake MARS3, Thermo Electron, GmbH) at 25 °C with an angular frequency range of 0.1–100 rad/s viscoelastic properties.

An appropriate amount of the hydrogel was drawn into a 1 ml syringe, and the hydrogel was patterned on the cardboard by squeezing the syringe plunger to verify its injectability.

### *In vitro* release of Dox from hydrogel systems

We took 1 ml of Alg-Dox-R837 hydrogel prepared according to the above method, placed it in a dialysis bag with a molecular weight of 5 kDa, and transferred it to 50 ml of 0.9% NaCl injection solution. At multiple time points, 2 ml of dialysate was removed and the corresponding optical density (OD) was measured at 484 nm using a UV spectrophotometer to calculate the release of Dox.

### Drug distribution in hydrogel drug delivery systems

After 1 week of adaptive feeding of C57BL/6 mice, melanoma cells named B16F10 cells (1*10^6^ cells per mouse), were subcutaneously inoculated on the right back. On the 7th day after inoculation, Alg, Dox, and Alg-Dox were injected into the peritumoral stroma. After 48 h, the tumor tissues of the mice in each group were collected, embedded in OCT, and the frozen tissues were serially sectioned at 5 μm with a cryostat, and the frozen sections were placed on glass slides. The penetration and distribution of Dox in it were observed by optical microscope and fluorescence microscope.

The paraffin-embedded tissue sections were stained with immunofluorescence by deparaffinization, antigen retrieval, permeation, and blocking in 5% serum albumin. Fluorescently conjugated primary antibodies were incubated overnight at 4°C to label the peritumoral stroma with α-SMA. Dox has spontaneous light. Nuclei were stained with DAPI for 10 min. The sections were examined under fluorescence microscope.

### Cell-killing of drug-loaded hydrogel to B16F10 cells

The proliferation-inhibitory effect of hydrogels on B16F10 was assessed by CCK8 assay analysis. B16F10 cells were seeded in 96-well plates at 3*10^3^ cells per well for 24 h. Discard the old medium. Add Dox loaded with gradient concentrations (0–100 μg/ml) sodium alginate hydrogel mixed with cell culture medium (10% drug loaded hydrogel, 9% fetal bovine serum, 81% DMEM medium, 1% double antibody), each concentration was set to three parallel wells, after a total of 24 h and 48 h incubation, PBS was added to wash once. Add 100 μL of 10% CCK-8 medium to each well, place in a cell incubator to incubate for 4 h and use a multi-mode microplate detection system to read the absorbance at 450 nm wavelength.

### Live and dead staining of cells

To visualize dead and live cells after different treatments, fluorescence images of cells were acquired by propidium iodide (PI) and acetoxymethyl ester (AM) staining. First, 5*10^5^ B16F10 cells were inoculated into confocal dishes and allowed for 24 h to adhere. Each group was administered according to Blank (without any treatment), Alg, Dox, Alg-Dox, Alg-R837, and Alg-Dox-R837groups, and incubated for 24 h. The cells were taken out and stained, and then placed under a confocal microscope (UltraVIEW VoX, PerkinElmer Instruments Shanghai Co., Ltd.) to observe and record at 405nm, 488nm and 563 nm.

### *In vitro* activation of dendritic cells

To verify the role of drug-loaded hydrogels in immunity. The expression of immune molecules (CD80, MHCII) in DC2.4 cells after direct co-incubation with drugs was studied by ultrasensitive nanoflow cytometry (Attune NxT, Thermo Fisher Scientific Co., Ltd.), and related inflammatory factors (IL-6, TNF-α) were quantified by ELISA.

In order to simulate the actual drug onset process and study the effect of B16F10 tumor cell apoptosis on the activation of immune response, a transwell system was also introduced. Blank group (top layer without any treatment, not even B16F10 cells), and B16F10 cells treated with Alg, Dox, Alg-Dox, Alg-R837, Alg-Dox-R837 for 24 h were placed in the dishes. The residues obtained of B16F10 cells by different treatment methods are placed in transwell insert, and DC2.4 cells were incubated in the transwell chamber. The expression of immune molecules was detected after co-incubating again for 24 h.

### Antitumor efficacy in mice

Adaptive rearing of C57/BL6 mice was subcutaneously inoculated with 1*10^6^ B16F10 cells on the right back for 1 week. When the tumor volume reached 100 mm^3^ at day 7, mice were randomly divided into six therapeutic groups (*n* = 6), which were recorded as control group, Dox group, Alg-Dox group, Alg-Dox-R837 group, aPD-L1 group and Alg-Dox-R837+aPD-L1 group. They were weighed every 2 days, and the tumor growth on the back was observed.

Then, mice were immunized with one dosage of 100 μL of saline or 100 μL of saline with Dox alone or 100 μL gel of Alg-Dox, or gel of Alg-Dox-R837, with or without Dox pretreatment by interstitial injection at day 0. A single dose of gel loaded Dox interstitial injection pretreatment was administrated 2 days before the first immunization and the concentration was equivalent to 2.5 mg/kg body weight according to previous report. After pre-treatment, mice were treated with aPD-L1 immunotherapy at a dose of 2 mg/kg every 3 days. 14 days later, the mice were euthanized, the tumors were dissected and weighed, the weight and size of the tumors were observed, and the serum was collected.

### *In vivo* anti-tumor efficacy evaluation

Tumor length and width were measured every 2 days for each treatment group, and tumor volume was calculated based on the equation 0.5× length × width^2^. Representative tumor tissues were photographed on day 15 after treatment. Mice were sacrificed when the tumor volume exceeded 1,500 mm^3^. The body weight of the mice was recorded every 2 days from the time of treatment.

After 14 days of administration, the tumor tissue, liver and kidney organs of mice in each group were collected, fixed with 4% paraformaldehyde, dehydrated with gradient ethanol, embedded in paraffin, sectioned (thickness 5 μm), stained with H&E, and observed diseases of organs under an inverted microscope.

### Detection by flow cytometry

After treatment, immune cells were isolated from tumor tissues or tumor draining lymph nodes of each group of mice (*n* = 6) for immune response analysis. Typically, tumor draining lymph node DCs were isolated using RPMI 1640, cell filters (70 μm, BD FALCON), and stained with anti-CD45, anti-CD83, anti-CD80, and anti-MHCⅡ. Finally, cells were washed once with cell staining buffer and analyzed by flow cytometry (BD FACS Calibur, United States).

Tumor-associated macrophages (TAM) and cytotoxic T lymphocytes (CTL) were isolated from tumor tissues using RPMI 1640, collagenases I and III (1 mg/ml), red blood cell lysis buffer, and cell filters (70 μm, BD FALCON). Then, each sample was divided into two samples for TAM and CTL analysis respectively. For TAM analysis, samples were stained with anti-CD45, anti-F4/80, anti-CD86, and anti-CD206. For CTL analysis, samples were stained with anti-CD3, anti-CD8, and anti-CD4. Then, cells were flushed with cell staining buffer and analyzed by flow cytometry (BD FACS Calibur, United States).

### ELISA for TNF-α and IL-6 level

Cell medium supernatant and serum were collected and the secreted levels of TNF-α and IL-6 were determined by ELISA according to the manufacturer’s instructions. Then, the absorbance values of the samples at 450 nm were recorded with a fluorescent plate reader (Perkin-Elmer).

### Statistical analysis

Data are shown as mean ± SD (standard deviation) with at least three independent replicates. Statistical analyses were performed using a paired/unpaired two-sample Student’s t-test with GraphPad Prism 9.0 software; P-value < 0.05 was considered as significant (ns, no significance; *: *p* < 0.05; **: *p* < 0.01; ***: *p* < 0.001).

## Results and discussion

### Preparation and characterization of Alg-Dox-R837

**FIGURE 1:**
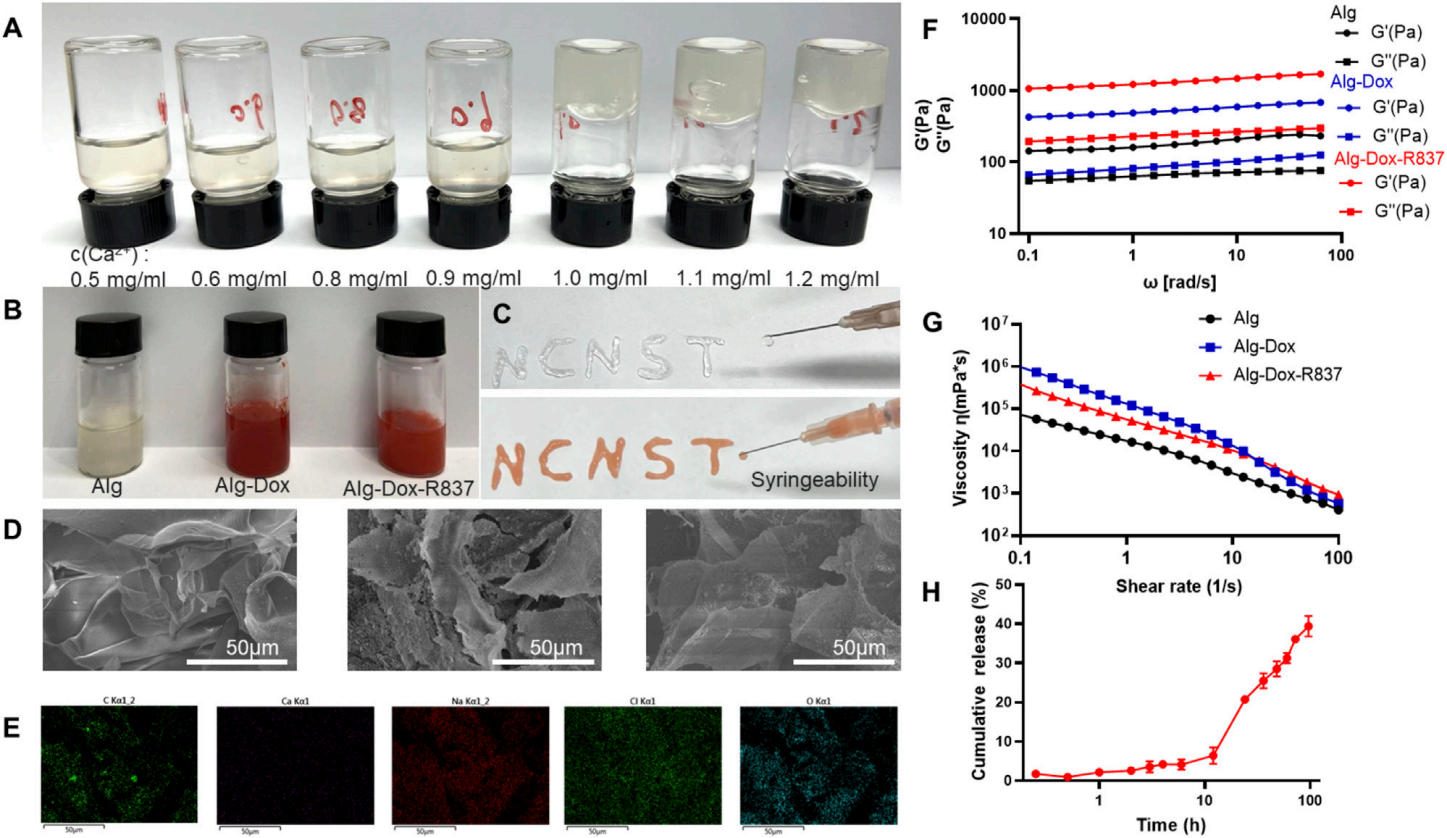
Characterization of Alg-Dox-R837 hydrogel **(A)** Gel formation with different Ca^2+^ concentrations; **(B)** Gel images with different components; **(C)** Alg-Dox-R837 formed “NCNST”; **(D)** Morphological characterization of hydrogel with different compositions (scale bar: 50 μm); **(E)** Elemental energy spectrum distribution of Alg-Dox-R837; **(F)** Loss modulus and storage modulus of Alg, Alg-Dox and Alg-Dox-R837; **(G)** Viscosity *versus* shear rate for Alg, Alg-Dox and Alg-Dox-R837; **(H)** *In vitro* sustained release profile of Dox in Alg-Dox-R837 gel.

The hydrogel for immunity inducing was prepared *via* simple mixing of alginate solution and Ca^2+^ solution. Based on the coordination between Ca^2+^ and carboxyl groups in alginate (Alg), Alg-Ca^2+^ hydrogel with an ionically cross-linked network could be obtained by simply mixing Alg solution with Ca^2+^ (Shu et al., 2021; Sun et al., 2021). The Ca^2+^ solution with different concentrations was added to the Alg solution (10 mg/ml) so as to determine the gel-forming conditions (Figure 1A). When the Ca^2+^ concentration was 1 mg/ml, a stable hydrogel system can be formed. The hydrophobic R837 was mixed with a low dose of Dox in the hydrogel solution. The optimal concentration of Ca^2+^ (1 mg/ml), Dox (0.5 mg/ml), and R837 (1 mg/ml) was chosen to synthesize Alg-Dox-R837 hydrogel (Figure 1B). Furthermore, the hydrogel exhibited excellent injectable properties, which can be easily extruded from a syringe and can be written into arbitrary patterns (e.g., NCNST logo) (Figure 1C). To verify the drug loading uniformity, the structure of the hydrogel was characterized by scanning electron microscopy, and the structure of the hydrogel did not change significantly after loading with Dox and R837, indicating that drugs were uniformly dispersed in the Alg-Ca^2+^ hydrogel system with strong loading capacity (Figure 1D). The energy spectrum shows the existence and good distribution of C, Ca, Na, Cl, and O element in the lyophilized Alg-Dox-R837 hydrogel, indicating that the combination of sodium alginate and calcium chloride for the preparation of the hydrogel was successful and feasible (Figure 1E). To examine the change in the mechanical properties of the hydrogel before and after the drug loading, the storage modulus (G′) and loss modulus (G″) of the hydrogel was measured. The results showed that with the incorporation of Dox and R837, both the G′ value and G″ value of the gel increased at the same time, indicating that the addition of drugs would increase the strength of the hydrogel. Meanwhile, the gel loaded with Dox and R837 showed more obvious shear thinning effect, which also demonstrated that the Alg-Dox-R837 hydrogel had excellent injectable properties (Figure 1G). What’s more, the experiment of Dox release was a necessary test to see whether the resulting material would be used in biomedical applications. The Dox release of Alg-Dox-R837 hydrogel system in 0.9% sodium chloride injection system was shown in Figure 1H. About 40% of Dox was released at 96 h. Overall, the release rate of Dox was decreased greatly and the release time of Dox is prolonged, so that it was convenient to realize the long-term release of low-dose amounts of Dox to kill tumor cells, then achieve the purpose of triggering ICD.

### Subcutaneous interstitial administration of gel to achieve tumor wrapped

**FIGURE 2:**
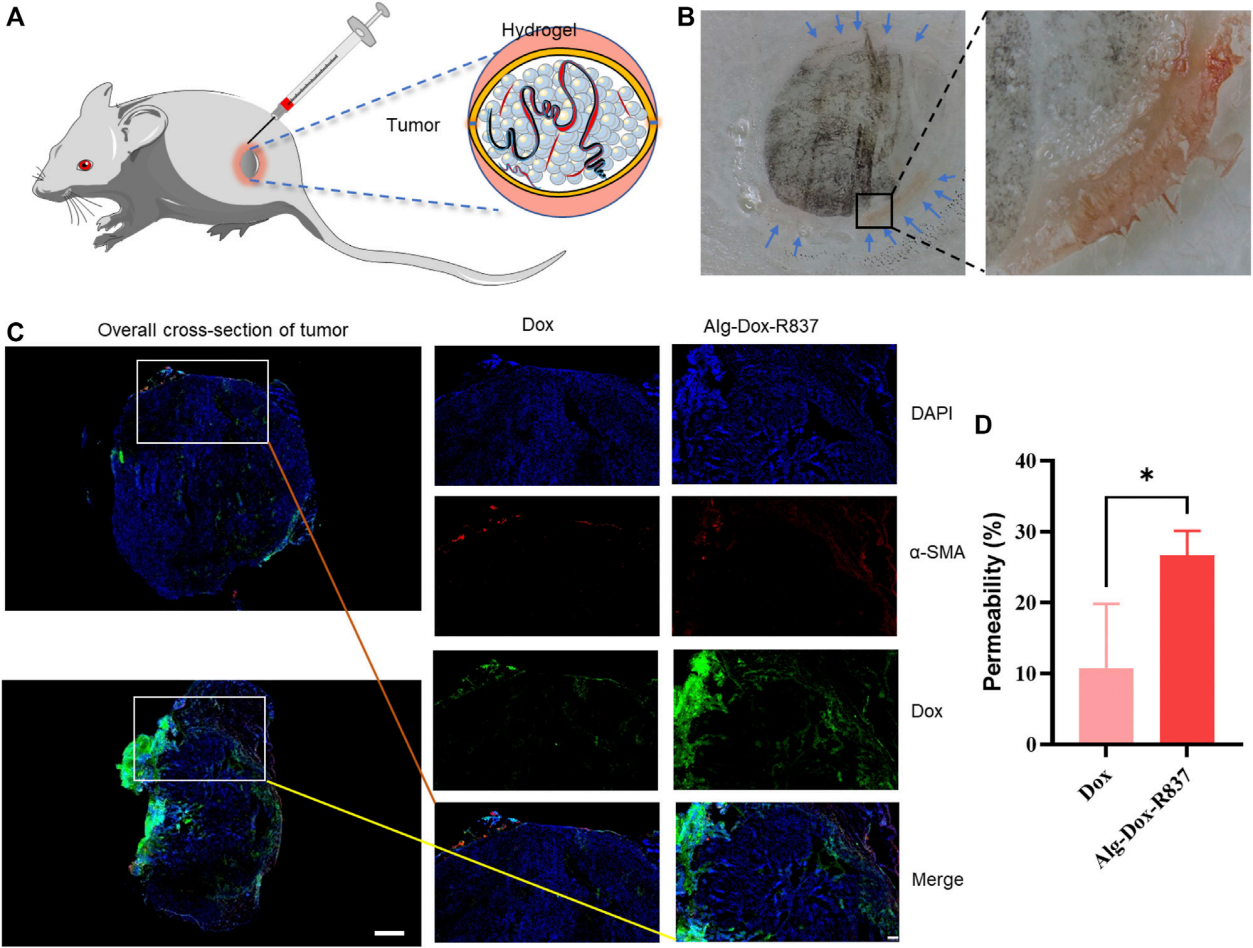
Subcutaneous interstitial administration of gel to achieve tumor wrap **(A)** Schematic diagram of interstitial injection of hydrogel to achieve tumor wrap; **(B)** Cryosection imaging of gel-wrapped tumor (black: melanoma, red: Alg-Dox -R837 gel); **(C)** Localized fluorescence imaging (scale bar: 200 μm) and overall cross-section imaging (scale bar: 1,000 μm) of Dox in tumors after treatments; **(D)** Permeability of tumor-to-stromal.

To verify the wrap of Alg-Dox-R837 hydrogel by subcutaneous interstitial injection around tumor sites (Figure 2A), we observed the frozen section of the tissue after the tumor stroma-wrapped hydrogel was wrapped in optimal cutting temperature compound (OCT). Alg-Dox-R837 hydrogel (red) was tightly distributed in the interstitial space around the tumor (black), achieving effective tumor wrap, which might block the formation of tumor blood vessels and the delivery of nutrients to the tumor (Figure 2B). To verify the release efficiency and infiltration of Dox in tumor tissues, α-SMA was subjected to fluorescence localization, which is positively and highly expressed in peritumoral tissue. Compared with pure Dox solution injected, the Alg-Dox-R837 hydrogel system exhibited a large amount of DOX infiltration and released into tumor tissue (Figure 2C), which would promote Dox to kill tumor cells effectively. The permeability of the drug was calculated from the fluorescence area. The results showed that the drug permeability of the free Dox group had a large error range, and the average permeability was 10.8%, which may due to the free Dox was metabolized faster in the body. In contrast, the error range in the Alg-Dox-R837 interstitial administration group was smaller, and the average permeability was 26.7%, indicating that Dox based on the gel carrier could enter the tumor tissue stably and had a higher level of Dox accumulated in the tumor tissues (Figure 2D).

### *In vitro* tumor cell killing and activation of dendritic cells

**FIGURE 3:**
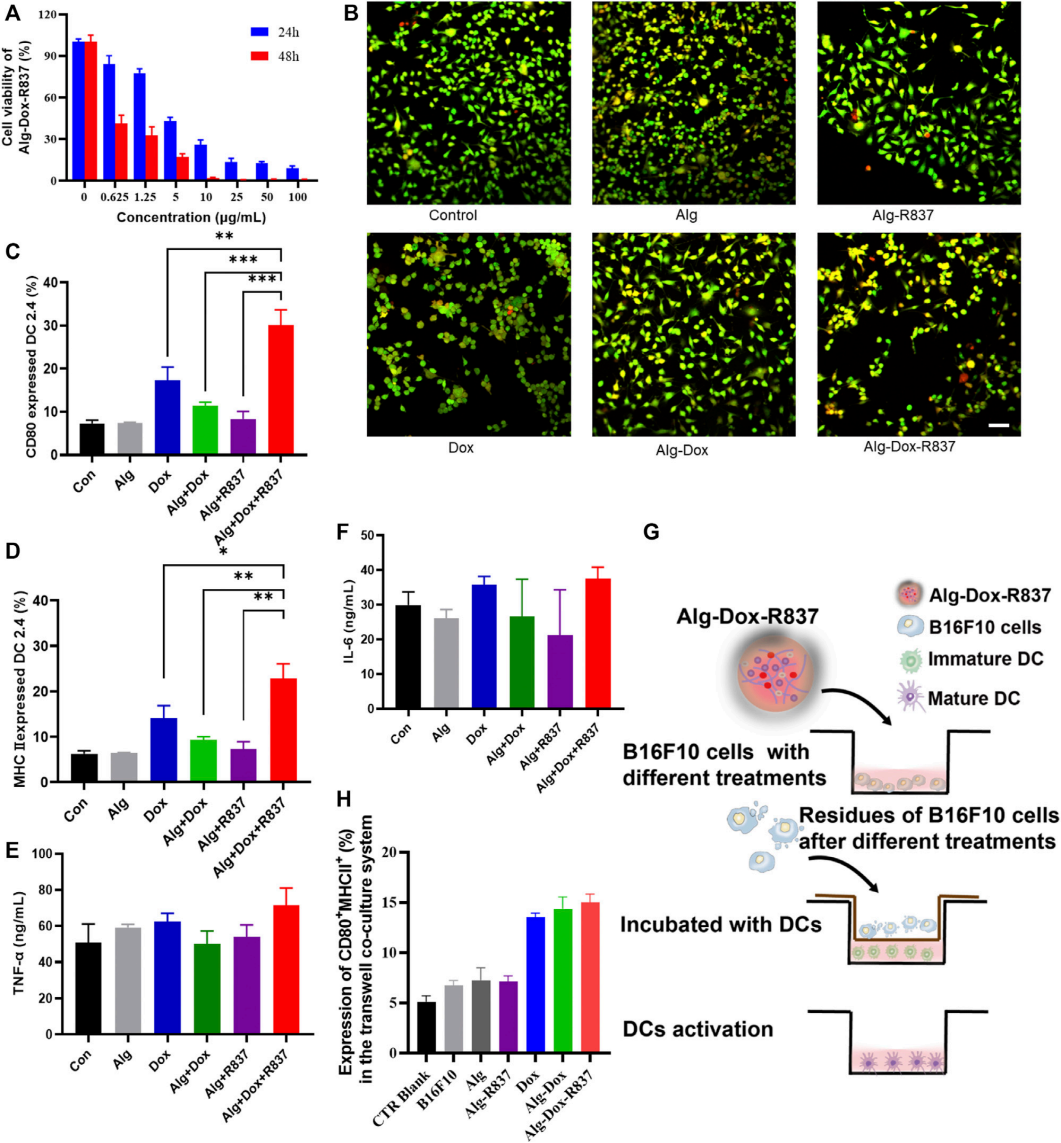
*In vitro* cell killing and activation of dendritic cells **(A)** Killing effect of Alg-Dox-R837 on B16F10; **(B)** Fluorescence images of dead and live cells after different treatments obtained by calcein AM and PI staining (scale bar: 70 μm); **(C,D)** The expression of DC surface markers CD80 and MHCII was assessed by flow cytometry; **(E,F)** The secretion of TNF-α and IL-6 in the culture supernatant of DC was measured by ELISA; **(G)** Schematic representation of B16F10 residue-induced DC activation after various treatments assessed using the transwell system. B16F10 cell residues with different treatments were placed in the transwell insert, and DC2.4 cells were cultured in the transwell chamber; **(H)** The expression of DC2.4 surface markers (CD80 and MHCII) in the transwell co-culture system was assessed by flow cytometry analysis.

The inhibitory effect of Dox-loaded hydrogel on melanoma cell B16F10 was evaluated by CCK-8 test. Based on the cell viability value of the group without Dox, the hydrogel containing different concentrations of Dox (0–100 μg/ml) showed obvious anti-tumor effects after cells were incubated for 24 h and 48 h. With the increase of Dox concentration, the inhibitory effect of the drug loaded hydrogel system on the proliferation of B16F10 cells became more obvious. After 24 h and 48 h incubation, the IC50 of the drug-loaded hydrogel system for B16F10 was 4.33 μg/ml and 0.13 μg/ml. The results showed that the drug-loaded hydrogel system could continuously and effectively inhibit the activity of B16F10 cells (Figure 3A).

To further verify the killing effect of the drug-loaded hydrogel on cells, we did cell live and dead staining. The results showed that Alg was safer for cells, and the proportion of apoptotic cells was low, indicating the biosafety of the hydrogel. The R837 adjuvant had no obvious killing effect on B16F10 cells. Dox has a certain killing effect on B16F10 cells, but due to the fast metabolism of Dox, the proportion of apoptotic cells is low. Alg-Dox and Alg-Dox-R837 showed a strong cell-killing effect due to their slow-release effect of Dox, while Alg-Dox and Alg-Dox-R837 showed no significant difference in cell-killing intensity (Figure 3B).

To verify the function of Alg-Dox-R837 in the immune response, costimulatory molecule expression and cytokine secretion in DC 2.4 cells were measured. We used flow cytometry to determine the expression of cell surface markers (MHCII and CD80) and used ELISA to quantify the levels of IL-6 and TNF-α in the supernatant of DCs after different treatments to verify the effect of DCs activation. The results showed that Alg itself could not increase the DC2.4 expression levels of MHCII and CD80, that is, Alg showed negligible effect on activating DCs. However, after adding an appropriate amount of R837 adjuvant and Dox, the expressions of MHCII and CD80 on the DC surface were significantly increased (Figure 3C&D), showing obvious DC activation. Mature DCs process phagocytic antigens and produce a number of pro-inflammatory cytokines to promote an immune response, which is presented to T lymphocytes, elicits humoral or cellular immunity. The results showed the levels of inflammatory factors (TNF-α, IL-6) in the cell culture medium were increased (Figure 3E&F). The Alg-Dox-R837 group had the most obvious activation effect on DCs.

At the same time, B16F10 was cultured in the upper layer of transwell plate, and after different drug treatments, the effect of B16F10 cells and their residues on DCs was observed (Figure 3G). The results showed that when DCs were in contact with B16F10 cells, the CD80 and MHCII expression of DC markers would slightly increase, indicating that DCs were activated to a certain extent. The Dox-treated groups, such as the Dox, Alg-Dox, and Alg-Dox-R837 groups, showed significant increases in the expression levels of surface markers MHCII and CD80 (Figure 3H). It showed that a small amount of killed tumor cells could effectively promote the maturation of DCs, thereby activating a strong immune response.

### *In vivo* antitumor efficacy

**FIGURE 4:**
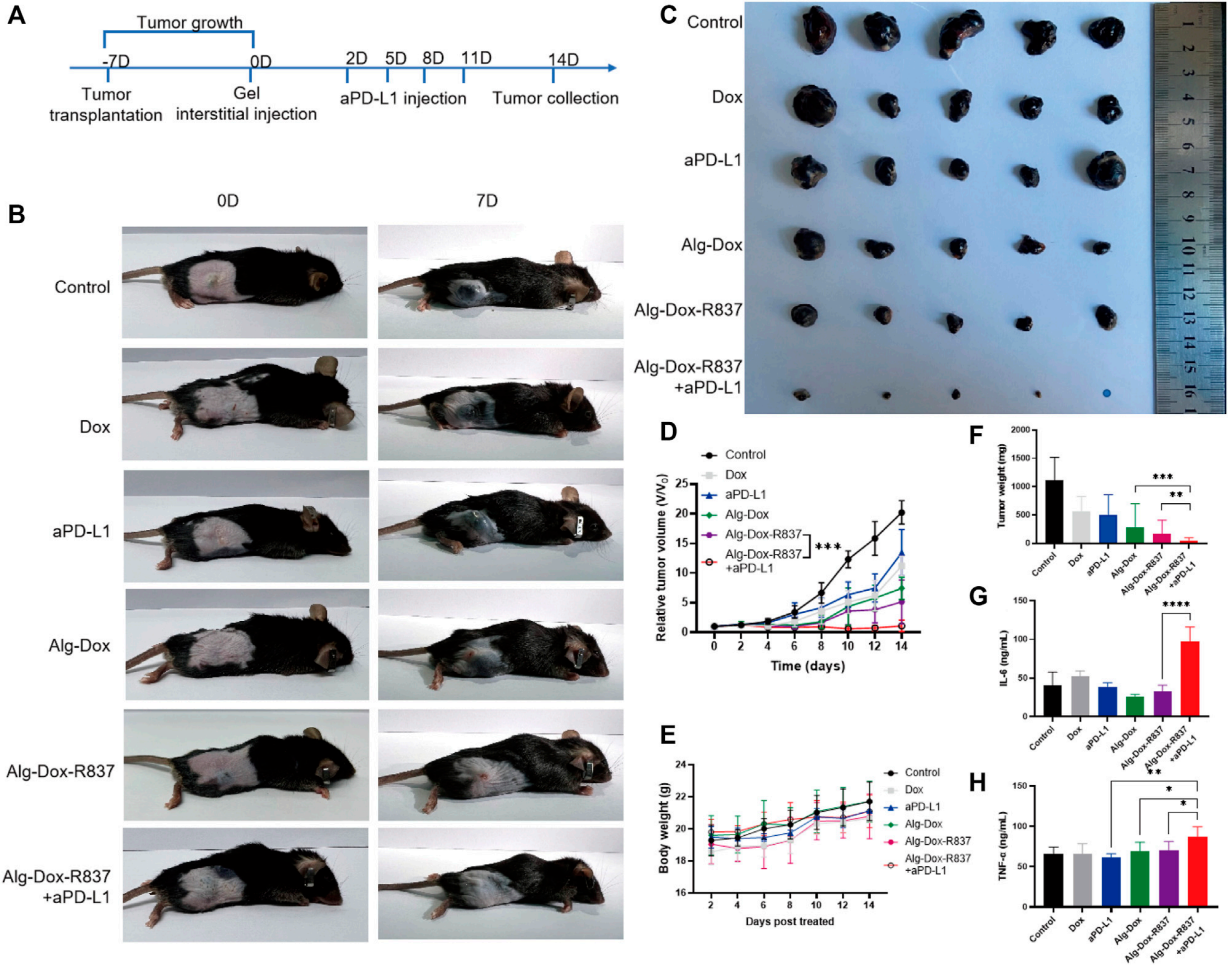
*In vivo* antitumor efficacy **(A)** Dosing schedule of interstitum-based chemo-immunotherapy *in vivo*; **(B)** Tumor image after 14 days of treatment in mice; **(C)** Image of isolated tumor after 14 days; **(D)** Treatment time curve of relative tumor volume during 14 days; **(E)** Curve of body weight with different treatments; **(F)** Weight of isolated tumor after 14 days of treatment; **(G)** Secreted IL-6 in mice serum after 14 days of treatment; **(H)** Secreted TNF-α in mice serum after 14 days of treatment.

In order to verify the antitumor effect of the Alg-Dox-R837 hydrogel system *in vivo*, we established a melanoma (B16F10) mouse model. After 7 days of modeling, the hydrogel was injected into the interstitium to wrap the tumor (Figure 4A). Following treatment, changes in tumor volume of mice were recorded daily (Figures 4B,D). The results showed that the tumor tissue of the mice in the untreated group grew rapidly. After interstitial administration of Dox alone, the growth of melanoma tumor tissue in mice was not significantly inhibited. This is because the metabolic rate of Dox is fast, and it is difficult to achieve significant therapeutic effect with lower frequency administration of Dox. At the same time, the single aPD-L1 administration group also did not exert a significant therapeutic effect. After administration of Alg-Dox, because Alg can slowly release Dox to kill melanoma, it makes up the disadvantage of the fast metabolism of Dox, which produces a certain inhibitory effect on tumor proliferation, and the volume growth of tumor tissue is slowed down to a certain extent. However, in the Alg-Dox-R837 group, the inhibitory effect on the tumor was more significant due to the immune activation effect of the R837 adjuvant. After the interstitial wrap of Alg-Dox-R837 hydrogel for 2 days, the injection of aPD-L1 through the lower extremity interstitium showed the strongest immune response and anti-tumor effect, and the tumor tissue volume of this group remained low level. After 14 days of treatment, the mice in each group were dissected, and it was found that the average melanoma volume of the mice in the Alg-Dox-R837+aPD-L1 group was the smallest, even some mice no longer detected tumor tissue, indicating that the interstitial immune response can effectively treat melanoma *in situ* (Figures 4C,F). The body weight changes of the mice in each group were monitored within 14 days. The results showed that the average body weight of the mice in each group increased generally within 14 days, indicating that the strategy of interstitial administration did not adversely affect the life quality of the mice (Figure 4E).

After 14 days of treatment, the serum of each group was collected for the detection of IL-6 and TNF-α. As shown in Figures 4G,H, the levels of serum inflammatory factors in the Alg-Dox administration group, Alg-Dox-R837 administration group and Alg-Dox-R837+aPD-L1 group increased, and the content of inflammatory factors in the serum of the mice was the highest in the Alg-Dox- R837+aPD-L1 administration group. It shows that the administration of interstitial hydrogel can significantly induce the immune response, achieving the purpose of safe and efficient tumor treatment.

**FIGURE 5:**
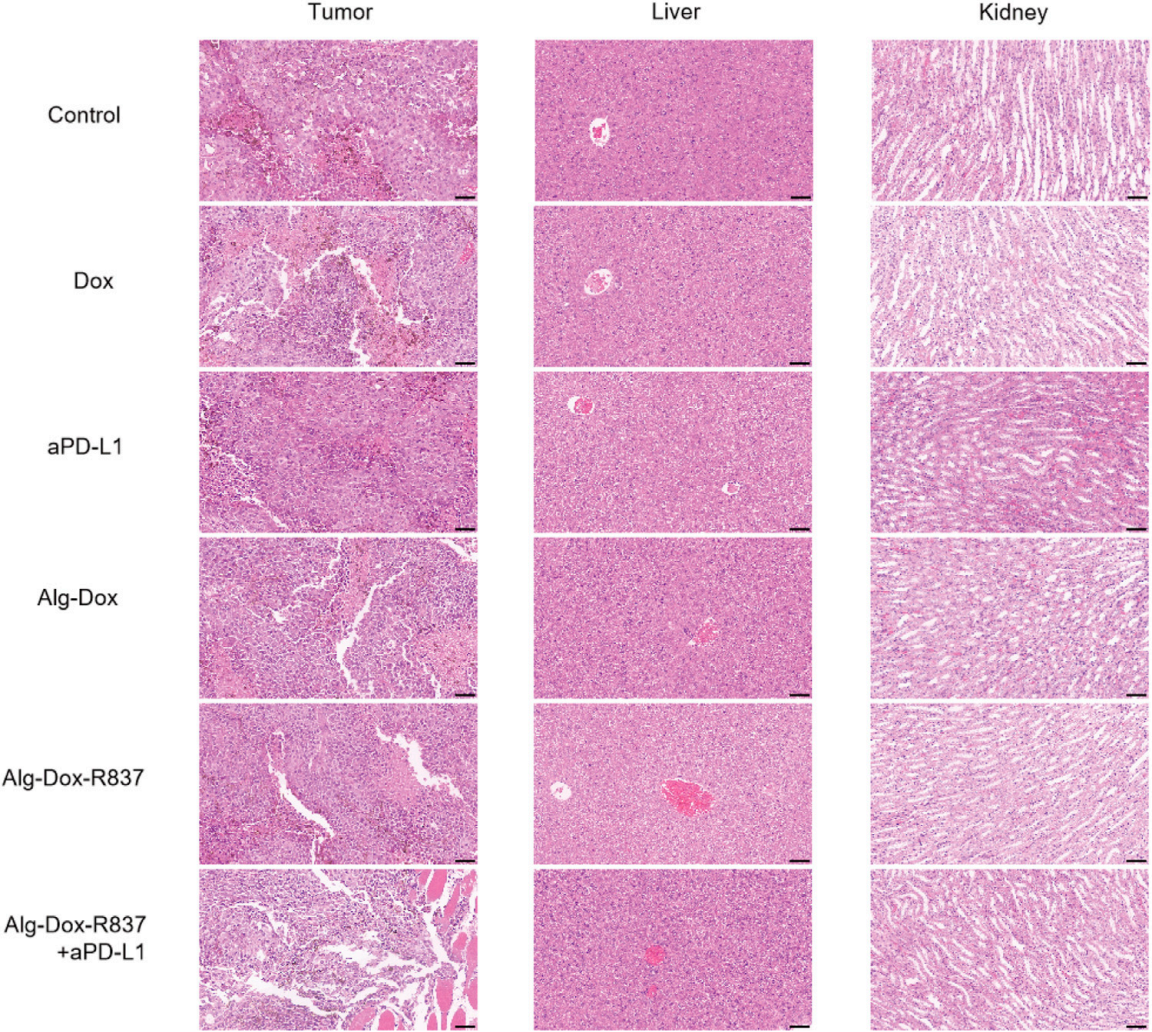
HE-stained images of major metabolic organs (liver and kidney) and tumor tissue after 14 days of drug administration in mice (scale bar: 50 μm).

After 14 days of administration, tumor tissue and major metabolic organs (liver and kidney) were collected for hematoxylin and eosin (H&E) staining (Figure 5). As shown in the figure, compared with the control group, slight necrosis of tumor cells was observed in the Dox, Alg-Dox, and Alg-Dox-R837 administration groups, indicating that the administration methods of Dox, Alg-Dox, and Alg-Dox-R837 have certain anti-tumor effect. The effect of single administration of aPD-L1 group was not obvious. However, extensive tumor tissue necrosis was observed in the Alg-Dox-R837+aPD-L1 group. In addition, the tumor tissue area in this group significantly shrank after administration, and the tumor tissue sections were surrounded by large pieces of normal muscle cells, indicating that the interstitial drug administration strategy can effectively inhibit the growth and invasion of tumor tissue. What’s more, there was no significant difference in pathological damage between the liver and kidney of the mice in administration groups and the control group, indicating that the interstitial administration had no obvious toxic or side effects on metabolic organs such as liver and kidney.

### Remodeling of the tumor microenvironment

**FIGURE 6:**
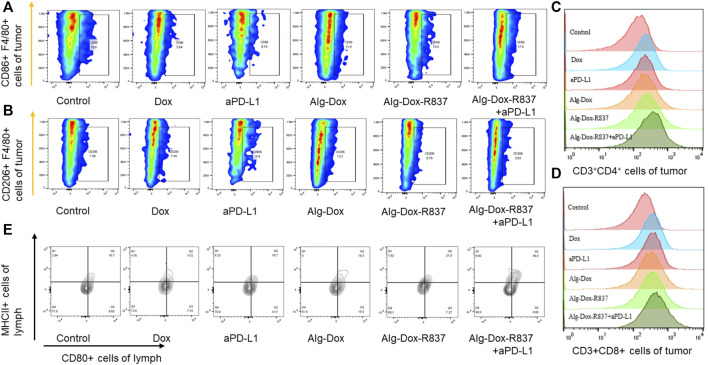
Remodeling of the tumor microenvironment **(A)** Changes in the number of M1-type macrophages (F4/80^+^CD86^+^); **(B)** Changes in the number of M2-type macrophages (F4/80^+^CD206^+^); **(C)** Changes in the number of helper T cells (CD3^+^CD4^+^) after treatment; **(D)** Changes in the number of killer T cells (CD3^+^CD8^+^) after treatment; **(E)** After treatment, the activation of DC cells in lymph nodes.

**FIGURE 7:**
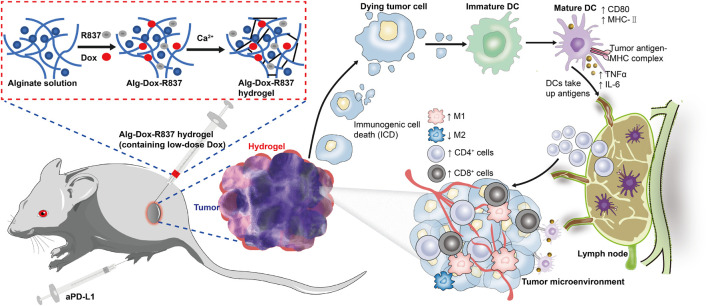
Schematic illustration of the mechanism of tumor immunotherapy by an engineered hydrogel through tumor interstitial wrap.

Altering the tumor microenvironment to suppress tumorigenesis is as important as direct tumor killing, and here we examined macrophage polarization in mice tumor tissues after administration by cellular markers (Cossarizza et al., 2021). As shown in the figure (Figures 6A,B), we observed that when only Dox, Alg-Dox, aPD-L1, and Alg-Dox-R837 were administered, the M1 or M2 cell marker values of macrophages in tumor tissues of mice (M1: F4/80^+^CD86^+^, M2: F4/80^+^CD206^+^) did not change significantly in each group, indicating that these administration groups had little effect on the polarization direction of macrophages. However, in the combined administration group of Alg-Dox-R837+aPD-L1, the M1 cell marker value increased, and the M2 cell marker value decreased, showing a more significant transformation of macrophages to the M1 type. At the same time, we detected and compared the changes of helper T cell (CD3^+^CD4^+^) and killer T (CD3^+^CD8^+^) cell markers in tumor tissues of mice in each group (Figure 6C&D). The results showed that the contents of CD4^+^ T cells and CD8^+^ T cell markers in the mice after administration increased to a certain extent, and the combined administration group that was administered Alg-Dox-R837+aPD-L1 detected the highest levels. It showed that the combined administration strategy of Alg-Dox-R837+aPD-L1 could significantly change the tumor microenvironment, promote the transformation of macrophages to the M1 type, and induce the increase of CD4^+^T and CD8^+^T cells in tissues, and a stronger T cell immune response would be stimulated for antitumor therapy. However, the analysis of the activation status of DC cells in the lymph nodes of mice (Figure 6E) showed that, except for the Alg-Dox-R837+aPD-L1 group, various administration methods had little effect on the activation status of DC cells. After administration of Alg-Dox-R837+aPD-L1, the amounts of cell markers MHCII and CD80 in CD83-expressing cells (dendritic cells) was significantly increased. It showed that this mode of administration inhibited the invasion and metastasis of the tumor wrapped, and enhanced antigen presentation to promote the maturation of DCs in the lymph nodes (Figure 7).

## Conclusion

In conclusion, a sodium alginate hydrogel system was prepared and the target drugs (Dox and R837) were incorporated into it. The results showed that the hydrogel system had good gel formation and good biocompatibility. After injection into the interstitial space around the tumor, it could slowly release Dox to kill tumor cells, activate and recruit more immune cells to generate an immune response with the help of R837, and finally produce a strong anti-tumor immune effect.

It has been reported that there are not negligible immune cells in the interstitium, which undertake important immune functions. However, there are few reports on the immune cell response and application of interstitial distribution. In this work, a hydrogel system was designed, embedded in the interstitial tissue and directly acting on the surrounding environment. The immune response of interstitial cells was successfully induced, the further development of melanoma was suppressed, and the tumor cells were successfully killed. It showed significance for the future interstitial administration to induce subcutaneous immune response, thereby reducing system toxicity and enhancing efficacy of therapeutic drugs.

## Data Availability

The original contributions presented in the study are included in the article/supplementary material, further inquiries can be directed to the corresponding authors.
